# Case Report: Isolated splenic hydatid cyst with negative serology managed by laparoscopic splenectomy

**DOI:** 10.3389/fpara.2026.1956525

**Published:** 2026-09-03

**Authors:** Amine Ben Safta, Salsabil Nasri, Wejih Dougaz, Hazem Alouani, Meriem Ksentini, Hichem Jerraya, Ramzi Nouira

**Affiliations:** 1 Department of Surgery B, Charles Nicolle Hospital, Tunis, Tunisia; 2 Universite de Tunis El Manar Faculte de Medecine de Tunis, Tunis, Tunisia; 3 Department of Pathology, Charles Nicolle Hospital, Tunis, Tunisia

**Keywords:** echinococcosis, hydatid cyst, laparoscopic splenectomy, negative serology, spleen

## Abstract

**Background:**

Hydatid disease caused by Echinococcus granulosus is a zoonosis endemic in many parts of the world. While hepatic and pulmonary involvements are common, isolated splenic hydatidosis remains extremely rare, representing less than 4% of all cases. Diagnosis can be further complicated by false-negative serological tests.

**Case presentation:**

We report the case of a 31-year-old female presenting with isolated left upper quadrant pain. Physical examination and baseline laboratory tests were unremarkable. Abdominal ultrasound and computed tomography (CT) revealed a well-circumscribed, homogeneous, hypodense oval mass (50×57×56 mm) at the upper pole of the spleen with a thin wall displaying a focal calcification. Hydatid serology was negative. Given the high suspicion of a hydatid cyst, pre-splenectomy immunizations were administered, and the patient underwent an exploratory laparoscopy. Intraoperative findings confirmed a splenic hydatid cyst, and a total laparoscopic splenectomy was performed. The postoperative course was uneventful. Histopathological examination confirmed the diagnosis.

**Conclusion:**

Clinicians should maintain a high index of suspicion for hydatid disease in patients presenting with cystic splenic masses in endemic areas, even in the presence of negative serology. Laparoscopic splenectomy is a safe and effective approach when strict hydatid safety measures are respected.

## 1 Introduction

Hydatid disease, or echinococcosis, is a parasitic infection caused by the larval stage of the tapeworm Echinococcus granulosus (McManus et al., 2003). It remains a major public health concern in endemic regions, such as the Mediterranean basin, the Middle East, and parts of South America (Karshima et al., 2022). The parasite typically enters the portal circulation, making the liver (60–70%) and lungs (20–30%) the most frequently affected organs (Sah et al., 2022).

Splenic involvement is rare, accounting for only 1.5% to 4% of all hydatid cases, and is usually associated with multi-organ dissemination (Akbulut et al., 2013). Although primary isolated splenic hydatidosis and laparoscopic management have been previously reported (Karabicak et al., 2009), the present case highlights an important diagnostic pitfall: hydatid disease may remain a diagnostic consideration despite negative serology when a splenic cyst occurs in an endemic setting (Okuş et al., 2013).

Herein, we describe, according to the CARE guidelines (Gagnier et al., 2013), this rare case of an isolated splenic hydatid cyst with negative serology in a young female, successfully managed via total laparoscopic splenectomy.

## 2 Case description

A 31-year-old female with no previous medical or surgical history presented to our department complaining of isolated, persistent left upper quadrant (LUQ) abdominal pain described as a sensation of heaviness, evolving over several weeks. She reported no history of fever, weight loss, trauma, or significant contact with dogs or livestock, and no relevant travel history beyond residence in an endemic area. She had received no prior treatment or intervention for this complaint.

On clinical examination, the patient was afebrile with a well-preserved general status and normal vital signs. The abdomen was soft and non-tender, and no palpable mass or splenomegaly was detected. The remainder of the systemic examination was unremarkable.

Routine laboratory investigations, including a complete blood count, liver function tests, and inflammatory markers, were within normal limits. Notably, no hypereosinophilia was observed.

## 3 Diagnostic assessment

**Figure 1:**
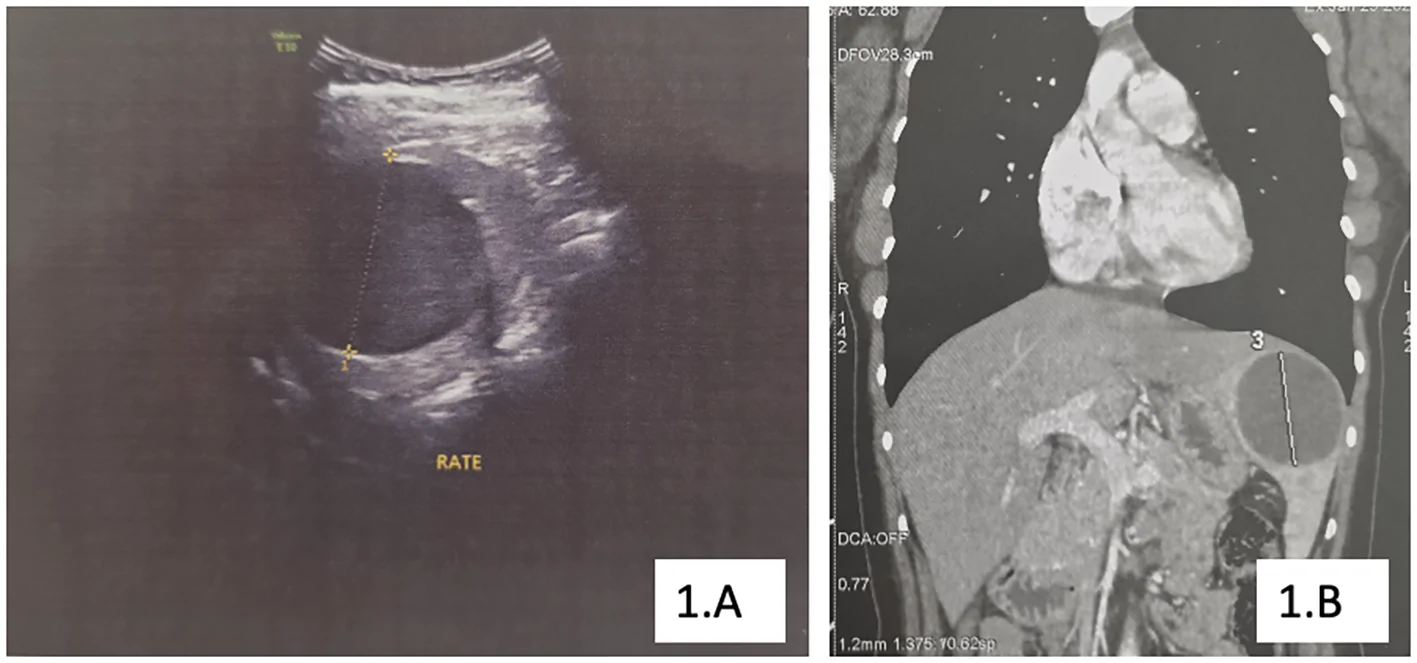
Preoperative imaging. **(A)** Abdominal ultrasound demonstrating a finely echogenic, well-circumscribed cystic lesion within the splenic parenchyma. **(B)** Contrast-enhanced abdominal CT showing a well-circumscribed, hypodense, homogeneous oval cystic mass measuring 50 × 57 × 56 mm at the upper pole of the spleen, with a thin, regular wall and a small peripheral calcification (arrow).

An abdominal ultrasound was performed as first-line imaging, demonstrating a finely echogenic cystic formation within the splenic parenchyma (**Figure 1A**). To better characterize the lesion, a contrast-enhanced abdominal computed tomography (CT) scan was obtained. The CT scan revealed a well-circumscribed, oval, hypodense, and homogeneous mass located at the upper pole of the spleen, measuring 50 × 57 × 56 mm. The cyst wall was thin and regular, displaying a small peripheral calcification. There was no internal septation, endoluminal budding, or contrast enhancement (**Figure 1B**).

Echinococcus-specific ELISA was negative. The test was not repeated, and no complementary immunoblot or quantitative antibody titre was available. Therefore, a low antibody titre or assay-related false-negative result could not be excluded.

Despite the negative immunological profile, the radiological features in an endemic context made a hydatid cyst the most probable diagnosis. An epidermoid cyst of the spleen, a post-traumatic or post-infectious pseudocyst, and a cystic neoplasm remained plausible differential diagnoses and were weighed against the imaging pattern, epidemiological context, and absence of a traumatic or infectious history.

The multidisciplinary staff decided on an exploratory laparoscopy both to confirm the diagnosis and to allow definitive surgical management in the same operative setting, given the low yield expected from image-guided biopsy in a suspected hydatid cyst (risk of anaphylaxis and peritoneal seeding).

**Table 1** summarizes the chronology of presentation, investigations, and management.

**Table 1:** Timeline of the episode of care, from initial presentation to six-month follow-up.

Timepoint	Clinical Event/Finding
Day 0	Presentation with isolated left upper quadrant heaviness; normal physical exam; normal CBC, LFTs, and inflammatory markers, no hypereosinophilia
Day 0	Abdominal ultrasound: finely echogenic, well-circumscribed splenic cystic lesion
Day 2	Contrast-enhanced abdominal CT: 50 × 57 × 56 mm hypodense, homogeneous cyst at the splenic upper pole with thin wall and focal calcification, no septa or enhancement
Day 5	Hydatid serology (ELISA for Echinococcus granulosus): negative
Day 7	Multidisciplinary discussion; decision for exploratory laparoscopy with a plan for total splenectomy if intraoperative findings confirmed hydatid disease
Day 7	Pre-splenectomy vaccination initiated (S. pneumoniae, N. meningitidis, H. influenzae type b)
Day 21	Exploratory laparoscopy confirming an intact upper-pole splenic hydatid cyst; total laparoscopic splenectomy performed with an endoscopic retrieval bag, no intraoperative spillage
Day 21–23	Uneventful postoperative course
Day 23	Discharge home
Day 28	Histopathology confirms laminated acellular membrane and germinal layer consistent with E. granulosus
Month 6	Outpatient follow-up: asymptomatic, no evidence of recurrence or dissemination, up to date on pneumococcal booster schedule

See **Table 1** in Section 3 (Diagnostic Assessment) above for the full timeline from presentation to six-month follow-up.

## 4 Therapeutic intervention, follow-up, and outcomes

In anticipation of a potential total splenectomy, the patient received the standard pre-splenectomy vaccination protocol against encapsulated bacteria (Streptococcus pneumoniae, Neisseria meningitidis, and Haemophilus influenzae type b), administered ahead of the planned procedure.

No perioperative albendazole or other systemic antihelminthic treatment was administered, as hydatid disease was not definitively established preoperatively because of the nonspecific imaging findings and negative serology. The diagnosis was suspected intraoperatively and confirmed histopathologically.

**Figure 2:**
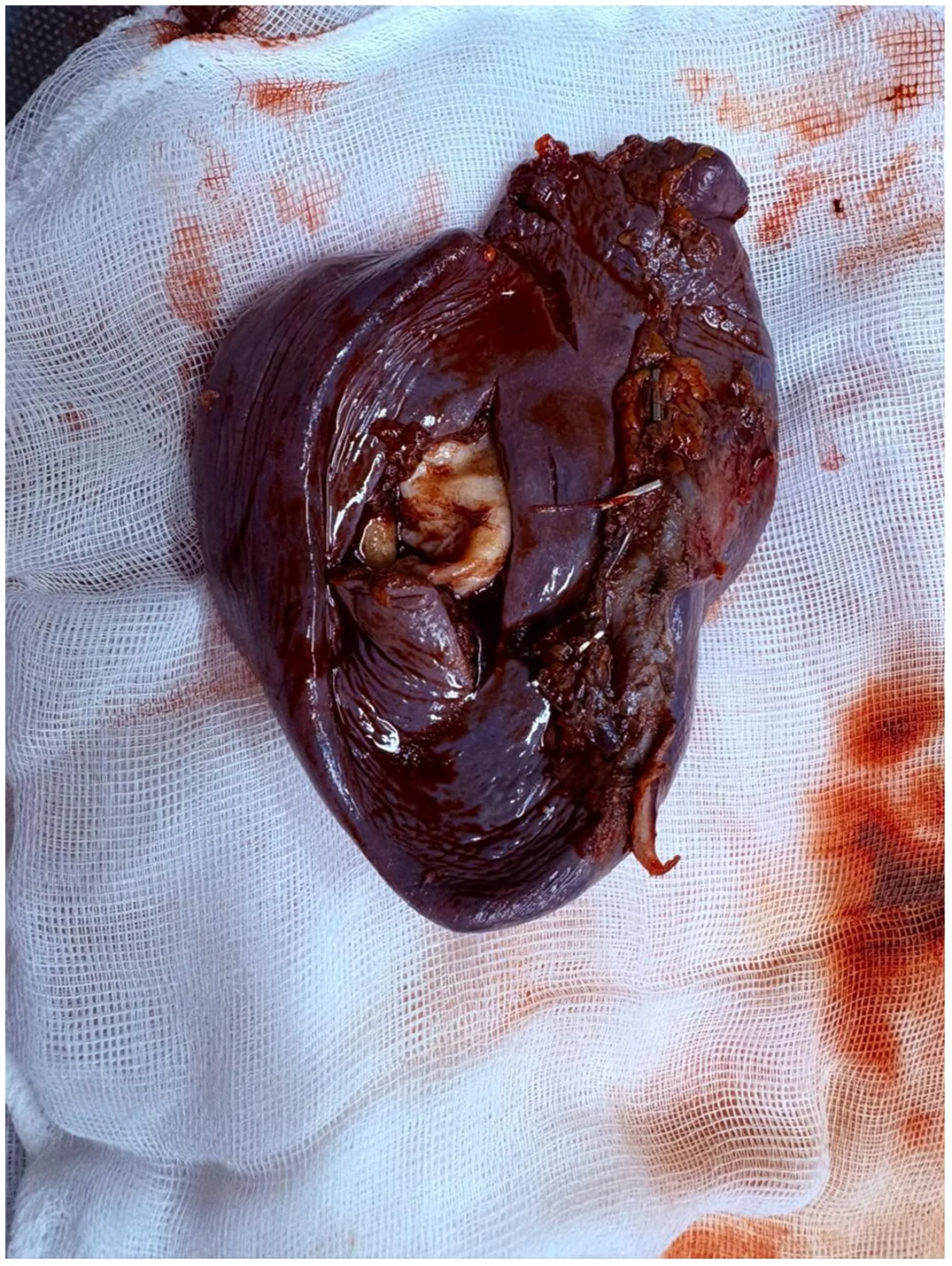
Intraoperative and gross specimen findings (composite). Surgical findings. Gross appearance of the resected spleen showing the unilocular hydatid cyst at the upper pole, containing typical hydatid fluid and characteristic membranes.

Intraoperative exploration revealed a cystic mass localized at the upper pole of the spleen, with an appearance highly consistent with a hydatid cyst. Careful dissection was carried out under low pneumoperitoneum pressure, taking strict precautions to prevent cyst rupture and intraperitoneal spillage, including early isolation of the splenic hilum and avoidance of direct instrument contact with the cyst wall. Due to the polar position and the risk of parasitic dissemination during partial resection, a total laparoscopic splenectomy was performed, and the specimen was extracted intact within an endoscopic retrieval bag (**Figure 2**).

**Figure 3:**
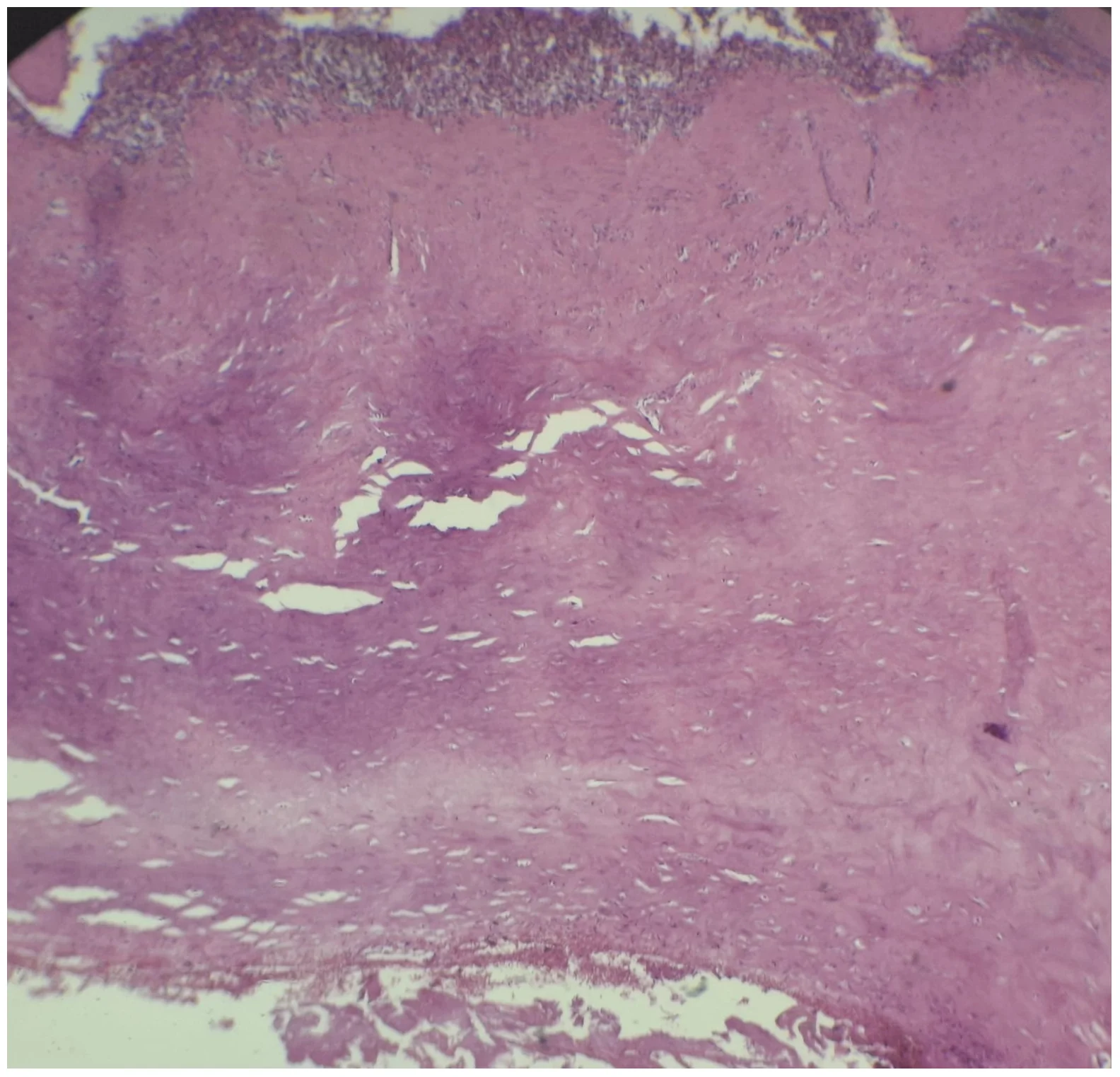
Histopathological examination. Microscopic examination of the resected surgical specimen showing the characteristic structural layers of the hydatid cyst wall, including the outer acellular laminated membrane and the inner germinal layer (Hematoxylin and Eosin stain, original magnification × 100), confirming the diagnosis of Echinococcus granulosus infection.

The postoperative course was uneventful, with no fever, bleeding, or signs of anaphylaxis. The patient was discharged on postoperative day two. Histopathological examination of the surgical specimen definitively confirmed the diagnosis of a splenic hydatid cyst, showing the characteristic acellular laminated membrane and germinal layer (**Figure 3**).

At six-month follow-up, abdominal CT showed no evidence of recurrent or disseminated hydatid disease. Hydatid serology was not repeated postoperatively.

## 5 Discussion

Primary isolated splenic hydatidosis is rare (Akbulut et al., 2013; Jallali et al., 2024a). The parasite typically reaches the spleen via systemic circulation after escaping the hepatic and pulmonary capillary filters, or retrograde through the portal venous system (Sah et al., 2022).

The clinical presentation is frequently indolent, as seen in our patient, who complained only of vague LUQ heaviness. The slow growth rate of the cyst allows the abdominal cavity to adapt, often delaying diagnosis until the cyst reaches a significant size or causes complications such as rupture, secondary infection, or hypersplenism (Dar et al., 2002).

A major learning point in this case is the negative hydatid serology. While serology is highly sensitive for hepatic hydatid cysts (up to 85–90%), its sensitivity drops significantly for splenic localizations (Jallali et al., 2024b). False negatives are frequently associated with intact cysts, dead or inactive parasites, or cysts with thick calcified walls that prevent antigen leakage into the bloodstream, thereby failing to trigger a detectable systemic immune response (Zhang and McManus, 2006; Brunetti et al., 2010; Griffin et al., 2014). Therefore, a negative serological test cannot rule out hydatid disease in endemic areas when imaging features are suggestive.

Radiologically, CT remains the modality of choice to define the morphology, exact anatomical relations, and presence of wall calcifications (Pedrosa et al., 2000). Differential diagnoses for splenic cystic lesions include true cysts (epidermoid), pseudocysts (post-traumatic or post-infectious), abscesses, and cystic neoplasms (Polat et al., 2003). In our case and based on the available ultrasound findings, the lesion could not be reliably classified according to the WHO-IWGE CE1–CE5 classification because characteristic daughter vesicles, detached membranes, or other stage-defining features were not documented. No molecular or genotypic confirmation was performed. The diagnosis was established histopathologically by identification of the characteristic laminated membrane and germinal layer.

Surgical intervention remains the cornerstone of management to prevent spontaneous or traumatic rupture, which can lead to life-threatening anaphylactic shock or secondary peritoneal hydatidosis (Dar et al., 2002). While spleen-preserving surgery (e.g., partial cystectomy or cyst enucleation) is appealing in young patients to avoid post-splenectomy sepsis, total splenectomy is often required for deep parenchymal or polar cysts, where the risk of rupture or major bleeding during dissection is high (Mejri et al., 2021). The polar location and size of the cyst in our patient favoured total splenectomy over a spleen-conserving approach.

The laparoscopic approach for splenic hydatidosis, once controversial due to dissemination risks, is now widely accepted as safe and effective when performed by experienced teams (Milosavljevic et al., 2019; Zhuoli et al., 2019). The use of a retrieval bag and avoidance of high pneumoperitoneum pressures are mandatory safety steps (Jani, 2014; Alqatta, 2026). Finally, appropriate pre-operative or post-operative immunization is mandatory when total splenectomy is performed, to mitigate the lifelong risk of Overwhelming Post-Splenectomy Infection (OPSI) (Arikanoglu et al., 2012).

The present case should not be considered unique because isolated primary splenic hydatidosis and laparoscopic treatment have been previously described (Semash and Voskanov, 2025; Zülfikaroğlu et al., 2026). Its main educational value lies in illustrating that negative serology does not exclude hydatid disease, particularly in an intact, partially calcified splenic cyst in an endemic setting.

Strengths and limitations: The strength of this report lies in the complete diagnostic and therapeutic pathway documented for a genuinely isolated, serology-negative splenic hydatid cyst, an uncommon combination. Its main limitation is that of any single case report: findings and outcomes cannot be generalized, and longer-term follow-up beyond six months would further strengthen confidence in the absence of recurrence.

Take-away lessons: (i) A cystic splenic mass in an endemic area should prompt consideration of hydatid disease irrespective of serology; (ii) negative serology reflects test sensitivity, not disease absence, particularly for calcified or extrahepatic cysts; (iii) laparoscopic splenectomy with a retrieval bag is a safe, definitive treatment when strict anti-spillage precautions are respected; and (iv) pre- or post-operative vaccination against encapsulated organisms is essential whenever total splenectomy is anticipated.

## 6 Patient perspective

The patient described the period between imaging and diagnosis as unsettling, particularly given the reassurance offered by the initial negative blood test, which she had hoped would exclude a parasitic cause. She reported that a clear explanation of why serology can be falsely negative, and of the reasoning behind proceeding to surgery despite this, helped her feel confident in the management plan. Following laparoscopic splenectomy, she described a swift recovery and was pleased with the cosmetic outcome and short hospital stay. At six-month follow-up, she reported feeling entirely well, had resumed her usual activities without limitation, and expressed satisfaction with the overall care pathway and communication she received throughout.

## 7 Conclusion

Isolated splenic hydatid cysts are rare clinical entities that can mimic other benign cystic lesions of the spleen, especially when serological tests are negative. Clinicians must rely on a combination of epidemiological context and strict radiological criteria. Laparoscopic total splenectomy is a safe, minimally invasive approach that offers definitive cure, provided that strict antiparasitic measures are implemented alongside mandatory prophylactic vaccinations.

## Data Availability

The raw data supporting the conclusions of this article will be made available by the authors, without undue reservation.
