# Supplementary material for: Case Report: Isolated splenic hydatid cyst with negative serology managed by laparoscopic splenectomy
Source: Front Parasitol. 2026 Sep 3;5:1956525. doi: 10.3389/fpara.2026.1956525 (PMC13582442; doi:10.3389/fpara.2026.1956525)
Supplement: Supplementary file 1 [file Supplementaryfile1.docx]

CARE Checklist of information to include when writing a case report

**Topic	Item	Checklist item description	Reported on Line**

**Title	1**	The diagnosis or intervention of primary focus followed by the words “case report”	Title

**Key Words	2**	2 to 5 key words that identify diagnoses or interventions in this case report, including "case report"	Abstract

**Abstract**

**(no references)**

**3a**	Introduction: What is unique about this case and what does it add to the scientific literature?	Background

**3b**	Main symptoms and/or important clinical findings	Case presentation

**3c**	The main diagnoses, therapeutic interventions, and outcomes	Case presentation

**3d**	Conclusion—What is the main “take-away” lesson(s) from this case?	Conclusion

**Introduction	4**	One or two paragraphs summarizing why this case is unique (**may include** reference**s**)	Introduction

**Patient Information	5a**	De-identified patient specific information	Case description

**5b**	Primary concerns and symptoms of the patient	Case presentation

**5c**	Medical, family, and psycho-social history including relevant genetic information	Case presentation

**5d**	Relevant past interventions with outcomes	Case presentation

**Clinical Findings**

**Timeline**

**Diagnostic Assessment**

**Therapeutic Intervention**

**Follow-up and Outcomes**

6 Describe significant physical examination (PE) and important clinical findings	Case presentation
7 Historical and current information from this episode of care organized as a timeline	Case presentation

**8a**	Diagnostic testing (such as PE, laboratory testing, imaging, surveys).	Diagnostic assessment

**8b**	Diagnostic challenges (such as access to testing, financial, or cultural)	Diagnostic assessment

**8c**	Diagnosis (including other diagnoses considered)	Diagnostic assessment

**8d**	Prognosis (such as staging in oncology) where applicable	Diagnostic assessment

**9a**	Types of therapeutic intervention (such as pharmacologic, surgical, preventive, self-care)	Thereupatic intervention

**9b**	Administration of therapeutic intervention (such as dosage, strength, duration)	Thereupatic intervention

**9c**	Changes in therapeutic intervention (with rationale)	Thereupatic intervention

**10a**	Clinician and patient-assessed outcomes (if available)	Follow-up and outcomes

**10b**	Important follow-up diagnostic and other test results	Follow-up and outcomes

**10c**	Intervention adherence and tolerability (How was this assessed?)	N/A

**10d**	Adverse and unanticipated events	N/A

**Discussion	11a**	A scientific discussion of the strengths AND limitations associated with this case report	Discussion

**11b**	Discussion of the relevant medical literature **with references**	Discussion

**11c**	The scientific rationale for any conclusions (including assessment of possible causes)	Discussion

**11d**	The primary “take-away” lessons of this case report (without references) in a one paragraph conclusion	Discussion

**Patient Perspective	12**	The patient should share their perspective in one to two paragraphs on the treatment(s) they received	Patient prespective

**Informed Consent	13**	Did the patient give informed consent? Please provide if requested . . . . . . . . . . . . . . . . . . . . . . . . . . . . . . . . . . . . .	**Yes**
